# A Textbook on the Principles and Practice of Surgery

**Published:** 1910-01

**Authors:** 


### BOOKS AND AUTHORS.

**A Textbook on the Principles and Practice of Surgery.** By **George Emerson Brewer,** M.D., Professor of Clinical Surgery in the College of Physicians and Surgeons, New York. Octavo, 908 pages, 415 engravings and 14 full-page plates. Lea & Febiger, Philadelphia and New York. 1909. (Cloth, $5.00; leather, $6.00, net prices).

This is the second edition of a one volume work of 900 pages, written by one of the leading surgeons and teachers of America. In his preface the author states that he has revised and completely rewritten his book and has added new material to the extent of two hundred pages. While this makes for an improvement over the former edition, a kindly criticism would be that it is yet too brief for a surgical textbook, that is supposed to cover the entire surgical field.

In his effort to be concise the author has to a considerable extent impaired the lucidness of his otherwise masterful treatise. A number of important subjects now occupying prominent places in surgical literature are either omitted or receive but meager mention.

The classification of the operation procedures are not in all cases detailed enough to give the beginners a thorough grasp of the subject. The general plan of the book however, is good. If Dr. Brewer in his next revision will elaborate along the lines already adopted, he will leave little to be desired in the way of a surgical textbook. J. A. R.

